# Synergistic energy harvesting and catalysis in B_2_SSe–MSSe (M = Mo, W) van der Waals heterostructures

**DOI:** 10.1039/d5ra07674a

**Published:** 2026-01-02

**Authors:** Basit Ali, Khadija Bashir, Yuanping Chen, Sailing He, M. Idrees, B. Amin

**Affiliations:** a Department of Physics, Abbottabad University of Science & Technology Abbottabad 22010 Pakistan binukhn@gmail.com +92-333-943-665; b School of Physics and Electronic Engineering, Jiangsu University Zhenjiang 212013 P. R. China; c National Engineering Research Center for Optical Instruments, Zhejiang University Hangzhou 310058 China; d School of Chemistry and Chemical Engineering, Shandong University Jinan 250100 China

## Abstract

In this study, we employed density functional theory (DFT) to investigate the structural, electronic, optical and photocatalytic properties of B_2_SSe–MSSe (M = Mo, W) van der Waals heterostructures (vdWHs). Due to the presence of different chalcogen atoms on either side of these Janus monolayers, multiple possible stacking patterns of B_2_SSe–MSSe heterostructures were constructed and analyzed. Their mechanical, thermal, and dynamical stabilities are confirmed *via* binding energies, Born criteria, *ab initio* molecular dynamics (AIMD) simulations, and phonon spectrum calculations. The weighted electronic band-structure analysis *via* both PBE and HSE06 functionals confirmed a type-II band alignment with an indirect bandgap. The intrinsic asymmetry of Janus materials introduces out-of-plane polarization and vertical electric fields, which facilitate efficient charge-carrier separation and migration, suppress recombination, and enhance photo-oxidation and visible-light absorption. Electrostatic potential profiles, charge density differences, and Bader charge analysis confirm interlayer charge transfer from the MSSe to the B_2_SSe monolayer, indicating p-type doping in the MSSe and n-type doping in the B_2_SSe of B_2_SSe–MSSe vdWHs. Higher carrier mobility in specific cases, verified *via* effective mass calculations, further promotes efficient charge transfer to the surface and reduces recombination across the interface of B_2_SSe–WSSe vdWHs, making them promising for light-detection and -harvesting applications. The imaginary part of dielectric function (*ε*_2_(*ω*)) indicating strong visible light absorption capacity. The valence and conduction band edge potentials were calculated to assess their photocatalytic suitability. The results demonstrate that B_2_SSe–MSSe vdWHs exhibit suitable band edges for overall water splitting at pH = 0, with the valence and conduction band edges straddling the redox potential window, enabling spontaneous oxygen evolution reaction (OER) and hydrogen evolution reaction (HER) under visible light irradiation. These findings underscore the potential of the B_2_SSe–MSSe (M = Mo, W) vdWHs for optoelectronic and photocatalytic hydrogen production applications and offer a valuable framework for designing the next-generation optoelectronic and photoharvesting devices.

## Abstract 2

In this study, we employed density functional theory (DFT) to investigate the structural, electronic, optical and photocatalytic properties of B_2_SSe–MSSe (M = Mo, W) van der Waals heterostructures (vdWHs).

## Graphical abstract

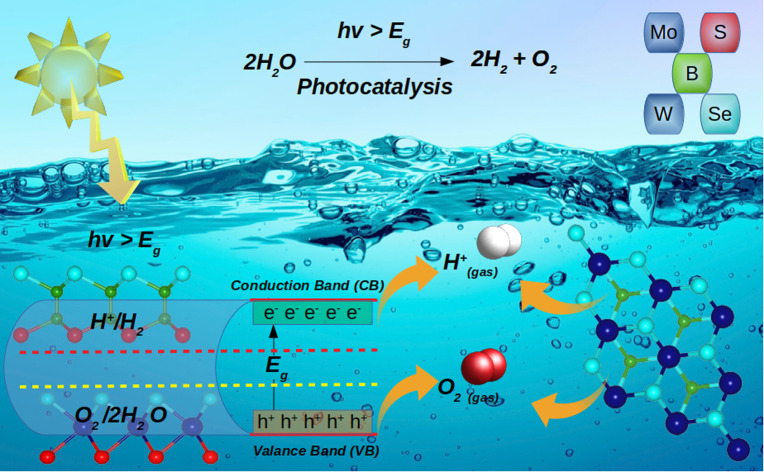

## Introduction

Photocatalytic water splitting is an emerging technique for producing renewable, green energy.^1–3^ Advanced catalytic materials play a pivotal role in various photocatalysis and photoelectrochemical processes,^4,5^ which use light energy to split water molecules into hydrogen and oxygen. When the photocatalyst is exposed to light in water, it absorbs photons and generates electron–hole pairs. The energy gap between the oxidation and reduction potential in water is 1.23 eV; therefore, materials with a minimum band gap of 1.23 eV are required to absorb light with energy equal to or greater than 1.23 eV .^6,7^ However, there still remain significant challenges for achieving full water splitting entirely driven by solar energy.^8,9^ Few photocatalysts are found to meet all the essential criteria for both the oxygen evolution reaction (OER) and hydrogen evolution reaction (HER). These criteria include an appropriate bandgap and band edge alignment, high carrier separation efficiency, strong visible-light absorption, spatially separated catalytic sites and strong redox capabilities.^10^ Effective separation of photogenerated carriers is hard to achieve with single-component photocatalysts, due to the recombination of the electron–hole pairs induced by the strong coulombic forces. Therefore, there is a critical need to explore stable and efficient photocatalysts that drive overall water splitting without the need for sacrificial agents or cocatalysts.^10–13^

In the last decade, two-dimensional (2D) materials, like transition-metal dichalcogenides (TMDs),^14,15^ layered double hydroxides (LDHs),^16^ and transition-metal carbides/nitrides (MXenes),^17,18^ have received significant attention in photocatalysis for water splitting due to their desirable physicochemical properties.^19,20^ However, the practical applications of most of these conventional photocatalysts remain limited by several factors, including poor optical absorption, high recombination rates of photoexcited electrons and holes, and inadequate redox potentials to facilitate the multistep proton- and electron-transfer processes required for water oxidation.^21,22^ Certain wide-bandgap semiconductors, like ZnO, g-C_3_N_4_ and TiO_2_, have band edge positions suitable for overall water-splitting enabling both the OER and HER, but their poor light absorption in the visible spectrum constrains their photocatalytic efficiency.^23–26^ Fe_2_O_3_ and Cu_2_O have bandgaps in the range of 2.0–2.2 eV and can effectively absorb visible light; however, they lack the necessary driving potential for overall water splitting.^27–29^ Additionally, in MoS_2_ monolayers, photogenerated electron–hole pairs tend to remain localized, leading to a high recombination rate that further limits photocatalytic efficiency.^30^

Ongoing research seeks to identify or design novel materials with broader energy absorption capabilities in the visible region to enable a strong ability for full water-splitting, or to tune existing materials to achieve this. Strategies like doping,^31,32^ alloying,^33^ strain engineering,^34^ electric field engineering,^35^ and controlled surface functionalization^36^ are used to tune the bandwidth and shift the absorption into the visible range. Vertical stacking of 2D materials in the form of van der Waals heterostructures (vdWHs) is the most effective way to tune the material properties, which can overcome these issues. vdWHs with type-II band alignment hold great potential for achieving efficient charge separation and reducing the electron–hole recombination rate, thus enhancing light-harvesting and photocatalytic applications.^37^ In the conventional type-II photocatalysts, the interfacial electric field plays a crucial role in the separation and recombination of photogenerated carriers.^38,39^

An inherent electric field arises due to the asymmetric structure in the Janus material,^40–43^ strengthening the efficiency of photogenerated-carrier separation and further influencing their recombination dynamics *via* the intrinsic dipole effect. This offers an additional mechanism for enhancing the photocatalytic performance for both the HER and OER.^44,45^ The first experimentally synthesized Janus TMDs (MoSSe and WSSe),^41,42^ with promising applications in spintronics and photocatalysis,^44,45^ have opened pathways to explore and fabricate novel Janus-based materials, like Janus group-III chalcogenides, Janus black arsenic–phosphorus, and Janus metal-carbides/nitrides.^46–48^ In the family of Janus group-III ternary chalcogenide monolayers, B_2_SSe has recently been explored *via* first-principles calculations.^49^ Janus MSSe (M = Mo, W) monolayers have also been extensively investigated for their unique structural asymmetry, tunable bandgaps, and potential in photocatalysis and optoelectronics.^50^ Both Janus MSSe (M = Mo, W) and B_2_SSe are indirect-band-gap semiconductors with intrinsic out-of-plane asymmetry, and their integration in a heterostructure offers great potential for tailoring band alignment, enhancing charge separation, and improving photocatalytic activity. However, the effects of the stacking configuration, interfacial chalcogen composition, and spin–orbit coupling on their optoelectronic and photocatalytic properties have not been systematically investigated.

Inspired by their unique structural asymmetry, strong intrinsic dipole moments and small lattice mismatch, this study employs first-principles calculations (see SI for detail) to investigate multiple stacking configurations in the form of B_2_SSe–MSSe (M = Mo, W) vdWHs, focusing on the role of interfacial chalcogen substitution (S/Se) in modulating electronic, charge-transfer and light responses. This study aims to provide a deeper understanding of the band alignment and spin–orbit coupling effects to assess the potential of B_2_SSe–MSSe (M = Mo, W) vdWHs for next-generation nanoelectronics, photocatalysis, and renewable energy harvesting applications.

## Results and discussion

**Fig. 1:**
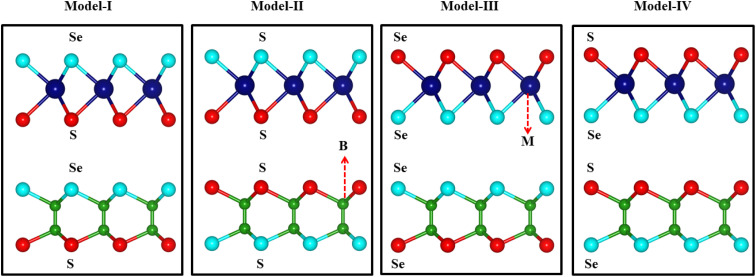
Model-I, -II, -III and -IV of the MSSe–B_2_SSe (M = Mo, W) vdWHs, where the blue/green balls indicate M/B atoms and the red/cyan balls indicate the S/Se chalcogen atoms.

**Fig. 2:**
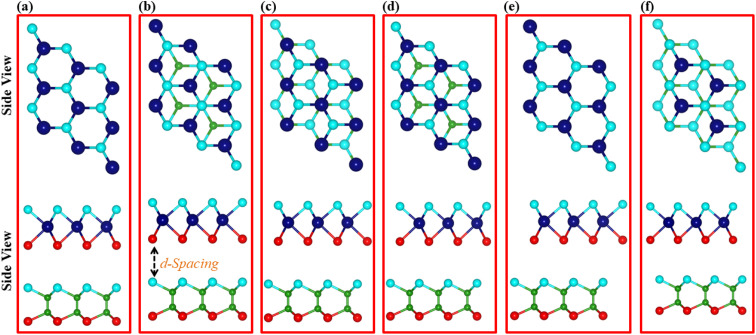
Different stacking configurations in model-I of the MSSe–B_2_SSe (M = Mo, W) vdWHs.

Previous studies on the monolayers of B_2_SSe^49^ and MSSe (M = Mo, W)^50^ provide a solid foundation for validating our current approach. The hexagonal lattice with a honeycomb structure of B_2_SSe and MSSe (M = Mo, W) monolayers is illustrated in Fig. 1. The hexagonal symmetry along with the given lattice mismatch (3.38(3.68)% of B_2_SSe with the MoSSe(WSSe) monolayer) allows the modelling of B_2_SSe–MSSe (M = Mo, W) vdWHs, where vdW interactions eliminate dangling bonds. The types of atoms at the interface of the vdWHs strongly affect the interfacial properties. Therefore, using the optimized lattice constant in the same hexagonal symmetry of B_2_SSe and MoSSe(WSSe) monolayers, we have modelled four stacking arrangements (model-I, model-II, model-III and model-IV) with different chalcogen atoms at the interface of B_2_SSe–MSSe (M = Mo, W) vdWHs; see Fig. 1. Six possible stacking configurations ((a)–(f)) are patterned for every single model (-I, -II, -III, and -IV) of the B_2_SSe–MSSe (M = Mo, W) vdWHs by shifting the atomic position of MSSe (M = Mo, W) on top of the B_2_SSe layer within the 1 × 1 unit cell of each layer, enabled by their small-scale lattice mismatch (<5%);^51^ see Fig. 2 (for model-I). In the case of stacking (a), the M(S) atom of the MSSe (M = Mo, W) layer is placed on top of the B(Se) atom of the B_2_SSe layer. In stacking (b), the S atom of the MSSe (M = Mo, W) layer is exactly settled on top of the Se atom of the B_2_SSe layer, while the M atom of the MSSe (M = Mo, W) and B atom of the B_2_SSe layer resides in the central position of the hexagon. In the case of stacking (c), the M atom of MSSe (M = Mo, W) is placed on top of the B atom of the B_2_SSe layer, while the S atom of the MSSe (M = Mo, W) layer and the Se atom of the B_2_SSe layer occupy the hollow sites of the hexagonal symmetry. In stacking (d), the M atom of MSSe (M = Mo, W) occupies the position on top of the Se atom of the B_2_SSe layer and the S(Se) atom of the MSSe(B_2_SSe) (M = Mo, W) layer is settled on the hollow position of the opposite layer. In stacking (e), the S(M) atom of the MSSe (M = Mo, W) layer is positioned on top of the B(Se) atom of the B_2_SSe layer. Meanwhile, in pattern (f), the S atom of the MSSe (M = Mo, W) layer is fixed on top of the B atom of the B_2_SSe layer and the M atom of the MSSe (M = Mo, W) layer occupies the hollow position in the hexagonal lattice site of the B_2_SSe layer. Patterns with similar arrangements are also repeated for model-II, -III and -IV of the B_2_SSe–MSSe (M = Mo, W) vdWHs.

Binding energies of the B_2_SSe–MSSe (M = Mo, W) vdWHs for all six ((a)–(f)) patterns in each model (-I, -II, -III and -IV) are calculated *via*:^51^*E*_b_ = *E*_B_2_SSe–MSSe_ − *E*_B_2_SSe_ − *E*_MSSe_, where *E*_B_2_SSe–MSSe_ is the total energy of the B_2_SSe–MSSe vdWHS, and *E*_B_2_SSe_(*E*_MSSe_) is the total energy of the of B_2_SSe(MSSe) isolated monolayer. The calculated binding energies along with their interlayer distances are listed in Table 1. The most stable stacking configuration is the one with the most negative binding energy and the shortest interlayer distance, indicating that stacking configuration (e) for model-I and model-III and stacking configuration (d) for model-II and model-IV are the most favorable stacking patterns among all six stacking configurations of these vdWHs. The obtained interlayer distances and binding energies are consistent with the range of previously calculated data,^52,53^ supporting that our systems are vdWHs. The variation in the stable stacking pattern is due to the distinct chalcogen atoms at the interface of the B_2_SSe-MSSe (M = Mo, W) vdWHs. The induced strain in the B_2_SSe-MSSe vdWHs due to the lattice mismatch of constituent layers alters the bond lengths, and hence may affect the stability of the patterns. Therefore, we re-optimized their lattice parameters, and the corresponding optimized lattice constants and bond-lengths are summarized in Table 2.

**Table 1:** Binding energies (*E*_b_, in eV) and inter layer distances (*d*, in Å) of stacking patterns ((a)–(f)) in model-I, model-II, model-III and model-IV of the B_2_SSe–MSSe (M = Mo, W) vdWHs

Stacking		(a)	(b)	(c)	(d)	(e)	(f)
**B**_**2**_ **SSe–MoSSe**							
Model-I	*E*_b_	−0.1548	−0.1561	−0.2009	−0.2083	−0.2085	−0.2048
	*d*	3.785	3.787	3.340	3.251	3.167	3.263
Model-II	*E*_b_	−0.1353	−0.1365	−0.1793	−0.1862	−0.1816	−0.1798
	*d*	3.799	3.781	3.242	3.145	3.313	3.254
Model-III	*E*_b_	−0.1583	−0.1585	−0.1976	−0.2107	−0.2119	−0.2023
	*d*	3.889	3.838	3.488	3.303	3.155	3.28
Model-IV	*E*_b_	−0.1428	−0.1437	−0.1804	−0.1937	−0.1906	−0.1821
	*d*	3.897	3.896	3.412	3.071	3.368	3.332

**B**_**2**_ **SSe–WSSe**							
Model-I	*E*_b_	−0.1608	−0.1621	−0.2081	−0.2175	−0.2178	−0.2127
	*d*	3.892	3.897	3.457	3.326	3.198	3.268
Model-II	*E*_b_	−0.1386	−0.1409	−0.1803	−0.1888	−0.1851	−0.1807
	*d*	3.758	3.764	3.433	3.156	3.168	3.218
Model-III	*E*_b_	−0.1634	−0.1642	−0.2044	−0.2187	−0.2201	−0.2092
	*d*	3.886	3.792	3.653	3.248	3.102	3.281
Model-IV	*E*_b_	−0.1464	−0.1473	−0.1838	−0.1968	−0.1945	−0.1854
	*d*	3.867	3.792	3.630	3.109	3.235	3.338

Optimized lattice constants (*a*, in Å) and bond lengths (M–X, B–X and B–B, in Å) of the B_2_SSe–MSSe (M = Mo, W) vdWHs, in model-I, model-II, model-III and model-IV

**Table:** 

B_2_SSe–MoSSe	*a*	Mo–S	Mo–Se	B–S	B–B	B–Se
Model-I	3.195	2.4040	2.5231	2.0210	1.7203	2.0759
Model-II	3.195	2.4044	2.5230	2.0197	1.7207	2.0771
Model-III	3.195	2.4060	2.5224	2.0210	1.7205	2.0767
Model-IV	3.195	2.4060	2.5228	2.0201	1.7203	2.0770

**Table:** 

B_2_SSe–WSSe	*a*	W–S	W–Se	B–S	B–B	B–Se
Model-I	3.2	2.4107	2.5299	2.0236	1.7212	2.0787
Model-II	3.2	2.4110	2.5297	2.0219	1.7205	2.0790
Model-III	3.2	2.4121	2.5290	2.0234	1.7211	2.0791
Model-IV	3.2	2.4125	2.5293	2.0224	1.7206	2.0791

We have further verified the thermal and dynamical stabilities of the most favorable stacking pattern (based on the calculated binding energy and interlayer distance) in model-I, model-II, model-III and model-IV of the B_2_SSe–MSSe (M = Mo, W) vdWHs.

**Fig. 3:**
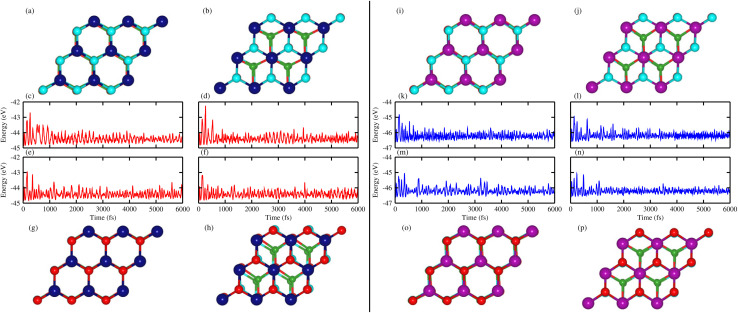
AIMD simulations of MoSSe–B_2_SSe ((a)–(h)) and WSSe–B_2_SSe ((i)–(p)). (a), (c), (i) and (k) are for model-I, (b), (d), (j) and (l) are for model-II, (e), (g), (m) and (o) are for model-III and (f), (h), (n) and (p) are for model-IV. (a), (b), (g) and (h) represent the geometrical structures and (c), (d), (e) and (f) represent the total energy fluctuations of the MoSSe–B_2_SSe vdWHs after heating, and (i), (j), (o) and (p) represent the geometrical structures and (k), (l), (m) and (n) represent total energy fluctuations of the WSSe–B_2_SSe vdWHs after heating.

The thermal stabilities of the B_2_SSe–MSSe (M = Mo, W) vdWHs are confirmed *via*Fig. 3, where the total energy as a function of time step (6000 fs) is plotted, with its corresponding geometrical structure after heating. The results demonstrate that the geometrical structure remains largely unchanged throughout the simulation. No considerable energy fluctuations were observed over time, and no bond breakages occurred in their final structures. The atoms only slightly vibrated and stayed near their equilibrium positions, except for the atoms in the B_2_SSe–MSSe vdWHs in model-IV, which show minor deviations from their equilibrium positions, indicating slight thermally induced distortions at 500 K. Hence, the overall results confirm that the structural framework of the B_2_SSe–MSSe vdWHs remains well preserved relative to its initially relaxed structure, which indicates that all considered vdWHs exhibit excellent thermal stability at 500 K, making them potential candidates for nanoelectronic device applications above room temperature.

**Fig. 4:**
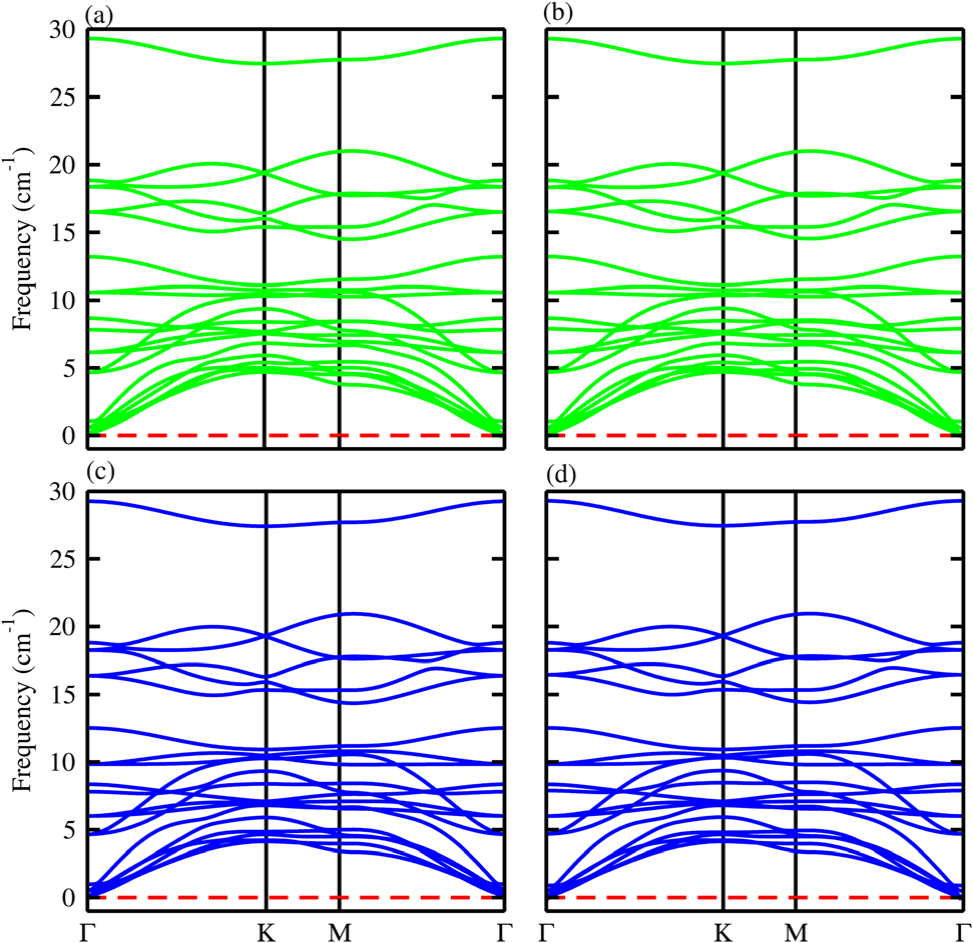
Phonon spectra of B_2_SSe–MoSSe (first row) and B_2_SSe–WSSe (second row) vdWHs. (a) and (c) are for model-I and (b) and (d) are for model-II.

The phonon spectra of the B_2_SSe monolayer in ref. 49 and the MSSe (M = Mo, W) monolayers in ref. 50 confirm the dynamical stability and collectively validate the feasibility of fabricating the B_2_SSe–MSSe vdWHs. The phonon spectra of the B_2_SSe–MSSe (M = Mo, W) vdWHs are calculated and illustrated in Fig. 4. The absence of imaginary phonon modes throughout the entire Brillouin zone (BZ) confirms the intrinsic dynamical stability of these systems. Each B_2_SSe–MSSe vdWH contains seven atoms per unit cell, with a total of 21 vibrational, 3 acoustic and 18 optical modes, as displayed in Fig. 4 for B_2_SSe–MoSSe and B_2_SSe–WSSe in both model-I and model-II. The acoustic branches include the longitudinal acoustic (LA), transverse acoustic (TA), and out-of-plane acoustic (ZA) modes. These findings confirm that the structures of the B_2_SSe–MSSe vdWHs are mechanically and dynamically robust, maintaining structural integrity without spontaneous collapse or distortion, making these materials promising candidates for nanoelectronics, optoelectronics and photocatalysis.^40,41^ The vibrational modes (both acoustic and optical) appear at energies similar to those of the constituent B_2_SSe and MSSe monolayers, indicating that the interlayer coupling is dominated by vdW interactions between these layers. A slight shift observed in the phonon mode positions, relative to the corresponding monolayers reported in ref. 49 and 50, is due to the induced strain during fabricating the vdWHs.

To assess the mechanical stability of the B_2_SSe–MSSe (M = Mo, W) vdWHs, we utilized the strain energy approach,^54^ wherein the elastic constants (C_*ij*_) are computed. In particular, we evaluated the in-plane elastic stiffness, *C*_11_, *C*_12_, and *C*_66_, with the Young modulus 
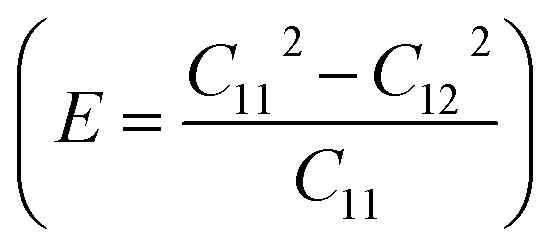
, bulk modulus 
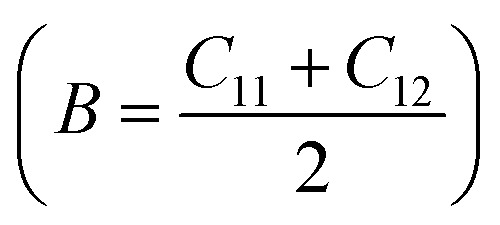
, shear modulus 
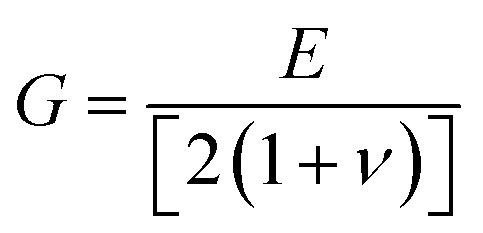
, Poisson ratio 
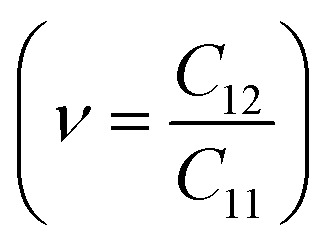
, and Pugh ratio (*B*/*G*), as listed in Table 3. The calculated elastic constants fulfill the Born criteria,^55–57^
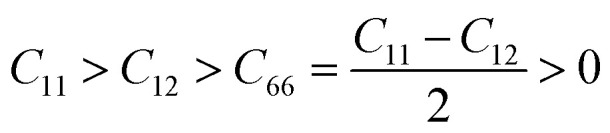
, confirming that these vdWHs are mechanically stable. Additionally, the higher value of *C*_11_ in Table 3 suggests that the B_2_SSe–WSSe structures are stiffer than B_2_SSe–MoSSe, hence showing more resistance to structural deformation. The bulk modulus (*B*) determines the out-of-plane stability and predicts the stability of the materials under pressure, while the Young modulus (*E*) measures the rigidity and in-plane mechanical stability under stress. High values of the Young modulus indicate the material is very stiff and resists deformation. Table 3 shows that both B_2_SSe–MoSSe and B_2_SSe–WSSe in model-II are more stable, stiffer and more resistant to structural deformation. The shear modulus (*G*) helps identify rigidity against shape change and measures the material respond to shear deformation. The calculated values in Table 3 show that the shear modulus is higher in model-III than in all other vdWHs. The Poisson ratio (*ν*) measures the ductile or brittle nature of a material. Generally, a higher value of the Poisson ratio (*ν* > 1/3) indicates ductility, while a lower value (*ν* < 1/3) suggests brittleness. A lower value of the Poisson ratio implies greater stability against shear stress.^58^ Similarly, the Pugh ratio is an important parameter widely used to predict the ductile or brittle behavior of materials; a critical value of 1.75 separates ductile (where *B*/*G* > 1.75) from brittle behavior (where *B*/*G* < 1.75).^59^ The Poisson ratio criteria (*ν* < 1/3) and Pugh ratio (*B*/*G* < 1.75) confirm the brittle nature of B_2_SSe–MSSe (M = Mo, W) vdWHs, and demonstrate strong stability against shear deformation; see Table 3. In these vdWHs, additional stress fields arise at the interface between the constituent layers of B_2_SSe–MSSe (M = Mo, W) vdWHs due to lattice mismatch and bonding asymmetry structures. These interfacial stress fields can induce dipoles that significantly alter the elastic properties, like stiffness and shear resistance, hence rendering these vdWHs more anisotropic compared to other materials like ZrSSe, SnSSe and ZrSSe/SnSSe vdWHs.^57^ The *C*_66_ parameters are also used to evaluate shear deformation within the basal plane, providing an assessment of the mechanical stability of the vdWHs. This parameter also helps in understanding energy dissipation mechanisms, such as interlayer sliding, which may occur under mechanical stress. The calculated values suggest that these vdWHs exhibit greater mechanical robustness due to interlayer van der Waals interactions and strain effects.

**Table 3:** Elastic constants (*C*_11_, *C*_12_ and *C*_66_, in N m^−1^), bulk moduli (*B*, in N m^−1^), Young moduli (*E*, in N m^−1^), shear moduli (*G*, in N m^−1^), Poisson ratios (*ν*) and Pugh ratios (*B*/*G*) of B_2_SSe–MSSe (M = Mo, W) vdWHs

	*C*_11_	*C*_12_	*C*_66_	*B*	*E*	*G*	*ν*	*B*/*G*
**MoSSe–B**_**2**_ **SSe**								
Model-I	348.652	76.121	136.266	212.387	332.033	136.266	0.218	1.559
Model-II	348.709	76.154	136.278	212.432	332.078	136.278	0.218	1.559
Model-III	347.857	74.904	136.476	211.380	331.727	136.476	0.215	1.549
Model-IV	347.722	75.743	135.989	211.733	331.223	135.989	0.218	1.557

**WSSe–B**_**2**_ **SSe**								
Model-I	362.701	75.963	143.369	219.332	346.791	143.369	0.209	1.530
Model-II	363.072	76.192	143.440	219.632	347.083	143.440	0.210	1.531
Model-III	362.277	75.367	143.455	218.822	346.598	143.455	0.208	1.525
Model-IV	362.556	75.756	143.400	219.156	346.726	143.400	0.209	1.528

**Fig. 5:**
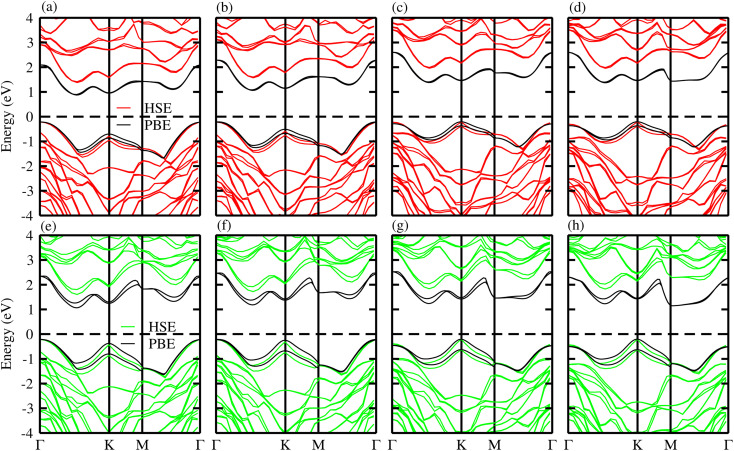
Band structures of the B_2_SSe–MoSSe (first row) and B_2_SSe–WSSe (second row) vdWHs. (a and e) For model-I, (b and f) for model-II, (c and g) for model-III and (d and h) for model-IV.

The choice of functionals has a significant impact on the determination of electronic properties. The common understanding in DFT is that semi-local functionals underestimate the bandgap, while hybrid functionals lead to better agreement with experiments; however, this is not universal, but rather depends on the considered materials.^60^ Therefore, both the PBE and HSE06 functionals are used for the in-depth examination of the electronic properties. The calculated band structures for the B_2_SSe–MSSe (M = Mo, W) vdWHs in model-I, -II, -III and -IV are depicted in Fig. 5, demonstrating that all models exhibit indirect-bandgap semiconducting behavior *via* both PBE and HSE06 methods. Interestingly, the bandwidth and band nature are highly sensitive to the choice of the interfacial atoms, particularly the substitution of the chalcogen atom (S or Se) in MoSSe and WSSe at the interface of the B_2_SSe–MSSe (M = Mo, W) vdWHs. In the case of model-I and model-II of B_2_SSe–MSSe, where the S atoms of MSSe are at the interface, the conduction band minimum (CBM) lies at the *Γ*–*K*-point, while the valence band maximum (VBM) is located at the *Γ*-point of the BZ. However, when the interface atom S is replaced with Se in model-III and model-IV, a transition in the band structure is observed: the VBM is relocated from the *Γ*-point to the *K*-point, and the bandwidth increases accordingly, as shown in Table 4. The bandgap values obtained using the HSE06 functional are significantly larger than those calculated at the PBE level. The inclusion of spin–orbit coupling (SOC) effects further reduces the bandgap due to the spin splitting of the VBM/CBM, as well as mirror symmetry breaking in the MSSe (M = Mo, W) and B_2_SSe monolayers, hence tuning the electronic band dispersion and enhancing relativistic effects.^61^ The indirect-bandgap nature of the B_2_SSe–MSSe (M = Mo, W) vdWHs indicates that photo-generated electrons and holes undergo indirect recombination, a process essential for energy harvesting and optoelectronic device performance.^51^ Moreover, in comparison to the individual monolayers, the B_2_SSe–MSSe vdWHs demonstrate noticeable bandgap (*E*_g_) modulation, underscoring the effectiveness of bandgap engineering in these systems.

**Table 4:** Bandgaps (PBE and HSE06, in eV), work functions (*ϕ*, in eV), potential drops (Δ*V*, in eV), carrier effective masses (
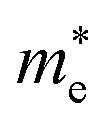
 and 
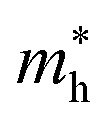
) and their ratios (Δ*D*), and redox potentials (*E*_CB_ and *E*_VB_) for the B_2_SSe–MSSe (M = Mo, W) vdWHs

	*E*_g-PBE_	*E*_g-HSE06_	*ϕ*	Δ*V*	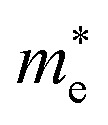	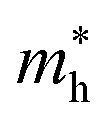	Δ*D*	*E*_CB_	*E*_VB_
**MoSSe–B**_**2**_ **SSe**									
Model-I	1.082	1.623	3.373	2.205	0.1549	0.2867	1.851	−0.157	1.465
Model-II	1.289	1.802	3.202	2.434	0.1542	0.2445	1.585	−0.246	1.555
Model-III	1.508	2.171	3.487	2.804	0.1544	0.1243	0.805	−0.431	1.739
Model-IV	1.596	2.193	3.345	3.197	0.1532	0.1226	0.800	−0.443	1.750

**WSSe–B**_**2**_ **SSe**									
Model-I	1.287	1.834	3.383	2.176	0.1325	0.2744	2.070	−0.177	1.656
Model-II	1.395	1.966	3.227	2.504	0.1319	0.2311	1.752	−0.243	1.722
Model-III	1.457	2.098	3.521	2.608	0.1316	0.0913	0.693	−0.309	1.788
Model-IV	1.341	2.079	3.387	2.929	0.1298	0.0908	0.699	−0.299	1.779

**Fig. 6:**
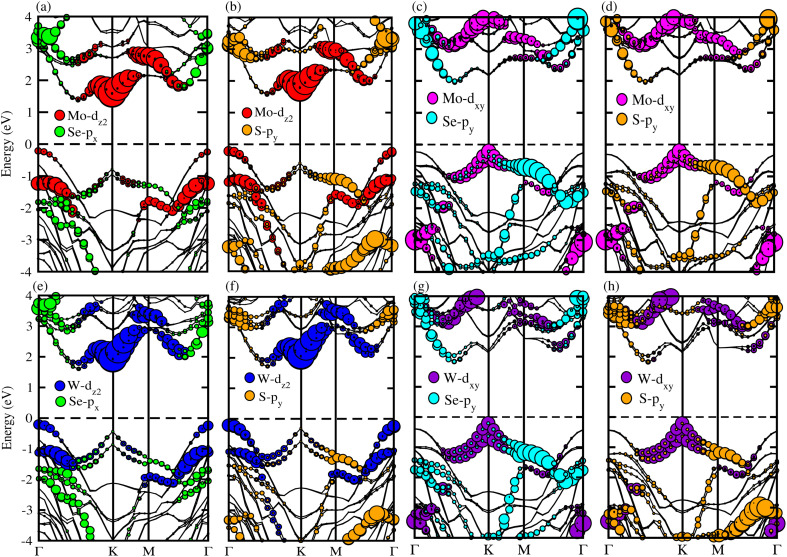
Orbitally-resolved (weighted) band structures of the B_2_SSe–MoSSe (first row) and B_2_SSe–WSSe (second row) vdWHs. (a and e) For model-I, (b and f) for model-II, (c and g) for model-III, and (d and h) for model-IV.

To gain deeper insight into the band structures of the B_2_SSe–MSSe(M = Mo, W) vdWHs, the orbital-resolved (weighted) band structures are calculated and depicted in Fig. 6. Both the CBM and VBM primarily originate from Mo(W)-d_*z*^2^_ orbitals and Se-p_*x*_ and S-p_*y*_ orbitals in model-I and model-II of the B_2_SSe–MSSe vdWHs; see Fig. 6(a, b, e and f). In contrast, for model-III and model-IV, the CBM is mainly derived from the Mo(W)-d_*xy*_ orbital, while the VBM is dominated by the Se(S)-p_*y*_ orbital, as illustrated in Fig. 6(c, d, g and h). These findings confirm the presence of type-II (staggered) band alignment in the B_2_SSe–MSSe (M = Mo, W) vdWHs, which facilitates charge carrier separation, specifically photoexcited electron–hole pairs migrating across different layers. Therefore, in the case of the B_2_SSe–MSSe vdWHs, the electrons migrate from the MSSe layer to the B_2_SSe layer, and holes in the reverse direction, thereby reducing the electron–hole recombination rate and creating built-in electric fields at the interface. This interlayer charge transfer mechanism plays a remarkable role in enhancing the performance of photocatalytic and photovoltaic applications.^62,63^

**Fig. 7:**
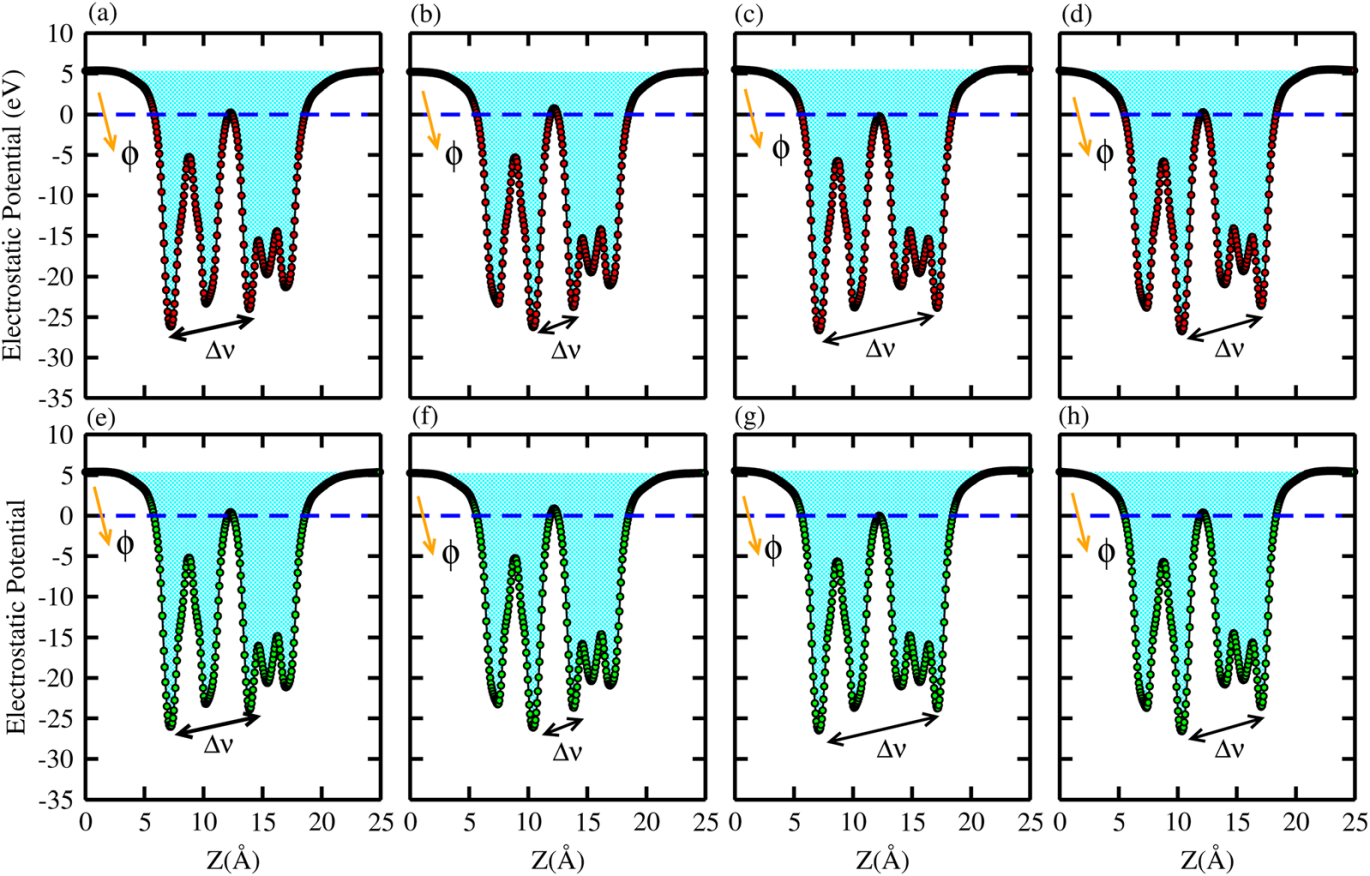
Electrostatic potential of the B_2_SSe–MoSSe (first row) and B_2_SSe–WSSe (second row) vdWHs. (a and e) For model-I, (b and f) for model-II, (c and g) for model-III, and (d and h) for model-IV.

The average electrostatic potential along the *z*-direction and charge density difference (CDD) calculations confirm the charge redistribution across the interface of the B_2_SSe–MSSe (M = Mo, W) vdWHs. These calculations provide both qualitative and quantitative insights into the interfacial charge transfer mechanisms. The electrostatic potential profiles in Fig. 7 indicate that the B_2_SSe(MSSe) layer exhibits a deeper(shallower) potential well, signifying that the electrons migrate from the MSSe to B_2_SSe layer at the interface of the B_2_SSe–MSSe vdWHs. This transportation of carriers reflects strong interlayer coupling, primarily arising from vdW interactions. The interfacial carrier separation introduces an intrinsic electric field across the interface, which is consistent with Cu_2_Se/SeIn_2_S^62^ and In_2_Se_3_/Sb heterobilayers.^63^ This intrinsic electric field not only enhances carrier mobility, but also modulates the work function (*ϕ*), thereby influencing the electronic behavior of these vdWHs. The calculated potential difference (Δ*V*) and *ϕ* values, summarized in Table 4, for model-I, -II, -III and -IV demonstrate the contrast between the excitonic characteristics of the individual monolayers and their corresponding vdWHs. The deeper potential well observed for sulfur (S) compared to selenium (Se) is attributed to their differences in electronegativity, which further influence the direction and extent of charge transfer across the interface. This disparity facilitates significant separation of photoexcited electron–hole pairs, thereby contributing to enhanced optoelectronic performance of these vdWHs. These results confirm the potential of the B_2_SSe–MSSe vdWHs for energy-harvesting and photo-response applications. Moreover, the strong interlayer coupling and interface-induced intrinsic electric field further reinforce their suitability for integration into advanced optoelectronic and photocatalysis applications.

**Fig. 8:**
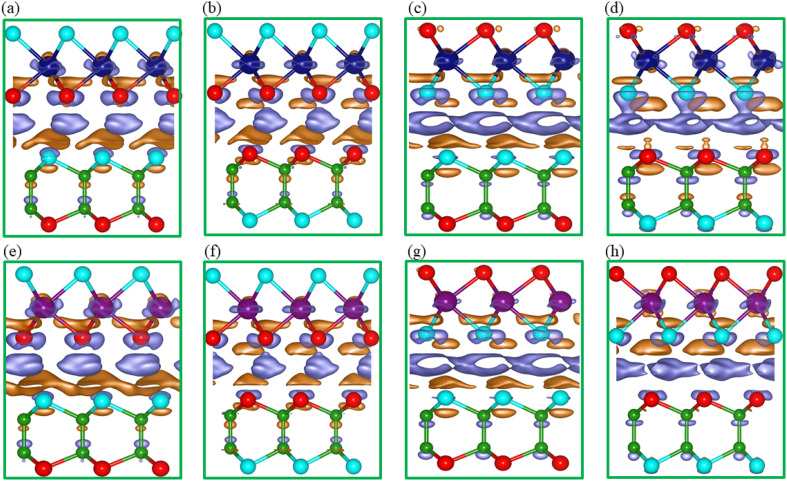
Charge density difference of the B_2_SSe–MoSSe (first row) and B_2_SSe–WSSe (second row) vdWHs for (a and e) model-I, (b and f) model-II, (c and g) model-III, and (d and h) model-IV.

To further analyze the interlayer charge transfer mapping and the induced intrinsic built-in electric field at the interface of the B_2_SSe–MSSe (M = Mo, W) vdWHs, the charge density difference (CDD) (Δ*ρ* = *ρ*_MSSe–B_2_SSe_ − *ρ*_MSSe_ − *ρ*_B_2_SSe_, where *ρ*_MSSe_, *ρ*_B_2_SSe_ and *ρ*_MSSe–B_2_SSe_ represent the charge densities of the isolated monolayers and their corresponding vdWHs) was calculated and plotted in Fig. 8. Charge accumulation(depletion) occurs around the B_2_SSe(MSSe) side, confirming the gain(loss) of electrons at the B_2_SSe(MSSe) interface; see Fig. 8. The visual representation of charge redistribution is highlighted by orange (accumulation) and silver–blue (depletion) regions. This redistribution of charge carriers confirms the existence of an intrinsic electric field arising from interlayer coupling and vdW interactions.

For better understanding of the interfacial charge transfer in the B_2_SSe–MSSe vdWHs, Bader charge analysis was performed. The results confirm the charge redistribution at the interface, where electrons migrate from the MSSe layer to the B_2_SSe layer and holes in the reverse direction, confirming the electron(hole) rich region around the B_2_SSe(MSSe) layer. In the case of B_2_SSe–MoSSe, the charge redistribution in model-I is approximately 0.0105 e(h), indicating that electrons transfer from the MoSSe to the B_2_SSe layer. In the case of model-II, about 0.005 e(h) are redistributed at the interface of the B_2_SSe–MoSSe in the same direction as in model-I. Similarly, in model-III and model-IV, 0.006 e(h) and 0.0018 e(h) are redistributed at the interface of the MoSSe(B_2_SSe) layer. A similar trend is also observed for the interface of the B_2_SSe–WSSe vdWHs, where the amounts of charge carriers redistributed are 0.0091 e(h) in model-I, 0.0041 e(h) in model-II, 0.0038 e(h) in model-III and 0.0008 e(h) in model-IV. These findings indicate that the MSSe(B_2_SSe) layer becomes p(n)-type doped and acts as a charge donor(acceptor). The degree of charge transfer varies with the stacking configuration, which underscores the critical role of the interfacial atomic arrangement, significantly modulating the amount of charge redistribution and overall performance.

After the absorption of sunlight, the recombination of photoexcited carriers plays an important role in determining the overall photocatalytic efficiency. However, in this context the formation of vdWHs not only modulates the band structure and band alignment, but also tailors the charge carrier dynamics due to induced strain within the constituent monolayers and the generation of an intrinsic electric field at the interface. It is essential to thoroughly understand the dynamical behavior of charge carriers at the interface.^61^ We further calculated the effective masses of carriers (electrons (
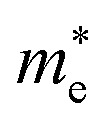
) and holes (
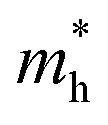
)) by using parabolic fitting of the VBM and CBM of these vdWHs for all models, *via*
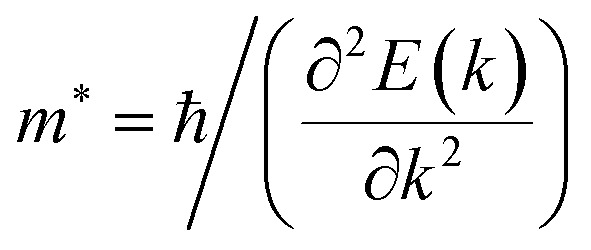
,^64^ along with their ratio *via*
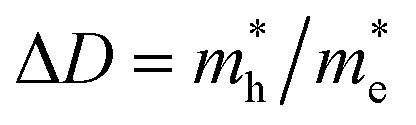
. Among the considered vdWHs, the results reveal a distinct variation in the effective mass behavior between the stacking models.

In model-I and model-II of B_2_SSe–MSSe, the effective mass of holes is greater than that of electrons, indicating a heavy-hole–light-electron system. This phenomenon facilitates efficient electron transport and implies slower hole mobility, hence promoting charge carrier separation and reducing recombination rates, ultimately enhancing photocatalytic efficiency.^50^ Both model-III and model-IV display the reverse trend, where the electron effective mass exceeds the hole effective mass, forming a light-hole–heavy-electron system. This shift in carrier dynamics may influence transport properties by making hole conduction dominant, thereby reducing the separation efficiency of photoexcited carriers under sunlight.^65,66^ In this case, the holes may escape faster while electrons lag behind; thus, it could increase recombination rates and potentially degrade the photocatalytic performance due to the lower mobility of the electrons. A smaller effective mass is associated with higher carrier mobility 
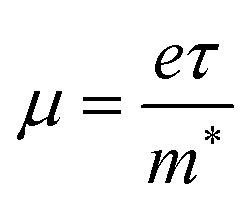
, a highly desirable feature for high-performance device applications.^61^ The effective masses for both electrons and holes as shown in Table 4 for B_2_SSe–WSSe in model-I and model-II are smaller than those in B_2_SSe–MoSSe, indicating a heavy-hole–light-electron system, which typically not only facilitates spatial charge separation, but also suppresses recombination, making a B_2_SSe–WSSe promising candidate for optoelectronic and photocatalysis applications. Meanwhile, in model-III and model-IV, the results indicate a heavy-electron–light-hole system, making such materials suitable for photovoltaic applications. The high p-type and n-type mobilities obtained from these vdWHs offer a unique strategy to tune the performance of optoelectronic, electronic, and energy harvesting devices. Δ*D* basically provides a pathway to determine the degree of spatial separation between photoexcited carriers. A higher value of Δ*D* signifies a larger disparity between the effective masses of holes and electrons, supporting more efficient charge separation.^64^ In the case of B_2_SSe–WS3Se, in model-I and model-II, the Δ*D* values are significantly larger than those of B_2_SSe–MoSSe, depending on the effective mass of electrons, as shown in Table 4. This suggests a more effective separation between electrons and holes in these systems, which leads to a reduction of carrier recombination rates and enhances further photogenerated charge carrier separation, thus making these vdWHs favorable for high-efficiency photocatalysis applications. Meanwhile, in the case of model-III and model-IV of B_2_SSe–MoSSe, the Δ*D* values are closer to 1, highlighting that the hole and electron mobilities are not significantly different.^64^ Variations in effective mass across the different stacking models underscore the sensitivity of charge transport behavior to interlayer atomic arrangements. These findings suggest that tailoring the stacking sequence in 2D vdWHs offers a feasible strategy to tune band alignment, carrier mobility, and recombination dynamics, crucial for maximizing the performance of electronic, optoelectronic, and photocatalytic applications.

**Fig. 9:**
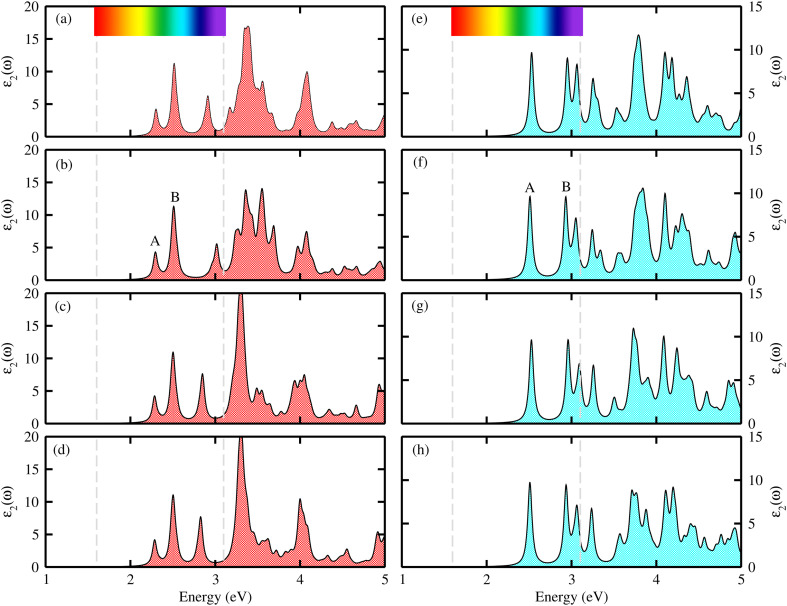
Imaginary part of the dielectric function of the B_2_SSe–MoSSe (left column) and B_2_SSe–WSSe (right column) vdWHs for (a and e) model-I, (b and f) model-II, (c and g) model-III, and (d and h) model-IV.

To investigate the absorption efficiency, we computed the imaginary part of the dielectric function (*ε*_2_(*ω*)) for the B_2_SSe–MSSe (M = Mo, W) vdWHs, as presented in Fig. 9. The range of the optical absorption is about 2 to 5 eV. Large absorption in visible region is observed in B_2_SSe–MoSSe due to a smaller band gap than that of B_2_SSe–WSSe, where a blue shift is observed for the latter. Higher carrier densities of vdWHs compared with the parent monolayers are associated with broadened optical absorption.^50^ A strong absorption capability for light in the visible spectrum (1.6 eV < *E* < 3.1 eV) is observed. Fig. 9 shows that the A(B) excitons correspond to the lowest energy optical transitions, with the peak positions observed at 2.305(2.515) eV for model-I, at 2.295(2.511)eV for model-II, at 2.285(2.504) eV for model-III and at 2.286(2.503) eV for model-IV of the B_2_SSe–MoSSe vdWHs. In the case of the B_2_SSe–WSSe vdWHs, the corresponding values are 2.530(2.952) eV for model-I, 2.5123(2.934) for model-II, 2.528(2.962) for model-III and 2.511(2.937) eV for model-IV. The binding energy of excitons is given by *E*_b-exciton_ = *E*_q_ − *E*_o_, where *E*_q_ is the quasi-particle band gap obtained from the GW approach and *E*_o_ is the energy of the first optical absorption peak observed from the BSE approach.^67^ The calculated *E*_b-exciton_ values for the B_2_Se–MoSSe vdWHs are 0.2106 eV for model-I, 0.362 eV for model-II, 0.64 eV for model-III and 0.622 eV for model-IV. Meanwhile, in the case of the B_2_Se–WSSe vdWHs, the values are 0.147 eV for model-I, 0.260 eV for model-II, 0.520 eV for model-III and 0.450 eV for model-IV. These values are notably smaller than those of isolated MoSSe and WSSe monolayers.^50^ Consequently, the absorption peak position and the corresponding exciton binding energies show significant variations when the chalcogen atom is shifted from S to Se at the interface, demonstrating the sensitivity of the optical response to interfacial charge redistribution. This observation underscores the superior light-harvesting capability of these vdWHs in the visible region. The inherent type-II band alignment facilitates efficient separation of photogenerated electron–hole pairs and suppresses their recombination rates, thus promoting enhanced charge carrier transport. These attributes suggest that B_2_SSe–MSSe vdWHs are promising candidates for photocatalysis, solar energy conversion, and optoelectronic applications.

**Fig. 10:**
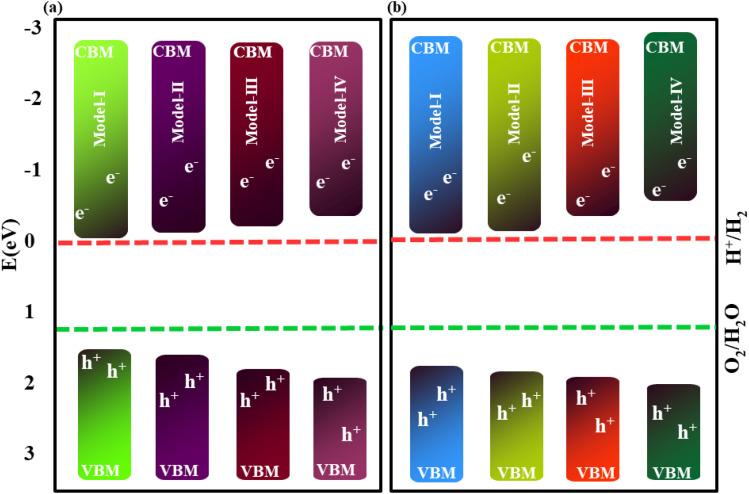
Photo-redox potentials for the vdWHs of B_2_SSe–MoSSe (a) and B_2_SSe–WSSe (b).

The position of the band edges plays a crucial role in assessing the photocatalytic performance of materials. An ideal photocatalyst should have a suitable band gap that straddles the redox potential of water. Specifically, the conduction band edge should lie above the reduction potential of H^+^/H_2_, activating the hydrogen evolution reaction (HER), while the valence band edge should lie below the oxidation potential of O_2_/H_2_O, activating the oxygen evolution reaction (OER). This alignment facilitates the design and selection of efficient photocatalysts that generate photoexcited electrons and holes with sufficient energy to drive the overall water splitting reactions. When a photon with energy greater than 1.23 eV is absorbed, electrons in the valence band are excited to the conduction band, leaving holes in the valence band. Therefore, the magnitude of the band gap must be equal to or greater than 1.23 eV to ensure effective solar light absorption and practical photocatalysis applications. The band alignments of B_2_SSe–MSSe with respect to the water redox potential at pH = 0 are presented in Fig. 10 and their corresponding redox potential values are summarized in Table 4. As shown in Fig. 10(a) for MoSSe–B_2_SSe and Fig. 10(b) for WSSe–B_2_SSe vdWHs, all the considered models exhibit energetically favorable band edge positions for both the HER and OER. The conduction band edges lie above the standard HER potential, while the valence band edges lie below the standard OER potential.

Hence, our predicted band edge positions indicate that B_2_SSe–MSSe vdWHs are promising candidates for full water-splitting, exhibiting spontaneous HER and OER under visible light irradiation, similar to ZnO–Al_2_SO heterostructures.^51^ In contrast, other Janus-based heterobilayers and trilayers, such as α-In_2_Se_3_/MSSe (M = W, Mo) and MoSSe–Ga_2_SSe, required additional treatments, including the creation of vacancies and the application of external voltages, to enhance photoelectrocatalytic performance.^68,69^ Thus, B_2_SSe–MSSe vdWHs possess superior intrinsic photocatalytic activity without the need for extrinsic modifications, along with enhanced structural stability, efficient charge separation, and tunable band alignment under visible light excitation at pH = 0, arising from their asymmetric Janus configuration and strong interlayer coupling. These exceptional properties indicate that B_2_SSe–MSSe vdWHs exhibit enhanced intrinsic stability and are more feasible for experimental realization compared to previously reported systems.^57,68,69^

## Conclusion

In summary, after optimizing the B_2_SSe–MSSe vdWHs in six possible stacking configurations, we investigated the electronic optical and photocatalytic properties of the most stable ones. AIMD simulations confirmed the thermal stability of these systems at 500 k, making them potential candidates for nanoelectronic device applications above room temperature. The phonon spectra and elastic constants (C_*ij*_) confirm that the structures of the B_2_SSe–MSSe vdWHs are dynamically and mechanically stable, maintaining structural integrity without spontaneous distortion, and exhibit a brittle yet shear-resistant nature. The heterostructures exhibit a staggered band alignment with an indirect band gap, tunable by chalcogen substitution at the interface, with SOC effects further reducing the bandgap due to the spin splitting of VBM/CBM. Charge transfer from the MSSe to B_2_SSe monolayer confirms an electron-rich region around the B_2_SSe layer, while a hole-rich environment is found around the MSSe layer, inducing p-type doping in MSSe and n-type doping in B_2_Sse, further enhancing efficient separation of charges. This also generates built-in electric fields that reflect strong interlayer coupling, making such materials suitable for photocatalytic and solar-cell applications. In model-I and model-II of B_2_SSe–MSSe, the effective mass of holes is greater than that of electrons, indicating a heavy-hole–light-electron system that implies slower hole mobility, while large Δ*D* values, especially in B_2_SSe–WSSe, favor photocatalysis. The reverse trend is observed in model-III and model-IV, where the electron effective mass exceeds the hole effective mass, forming a light-hole–heavy-electron system and making hole conduction dominant over electron conduction. The Δ*D* values of B_2_SSe–MoSSe are closer to 1, highlighting that the hole and electron mobilities are not significantly different, making these materials suitable for photovoltaic applications. The imaginary part of the dielectric function (*ε*_2_(*ω*)) calculations (GW + BSE) shows strong absorption capability of light in the visible spectrum (1.6 eV < *E* < 3.1 eV) with exciton binding energies that are highly sensitive to the interfacial chalcogen composition. Hence, the absorption peak positions show significant variations when the chalcogen atom is changed from S to Se at the interface, indicating enhanced light-harvesting capability. The band edge positions of the B_2_SSe–MSSe vdWHs confirm that they straddle the redox potentials for water splitting at pH = 0, enabling spontaneous hydrogen evolution reaction (HER) and oxygen evolution reaction (OER) under visible light irradiation. Overall, B_2_SSe–MSSe vdWHs combine optimal charge separation, tunable optoelectronic properties and favorable band alignment, making them a most promising candidate for high-performance photocatalysis and solar energy-conversion applications. This work provides insightful guidelines for designing Janus vdWHs through bandgap engineering and interfacial charge regulation.

## Conflicts of interest

There are no conflicts to declare.

## Supplementary Material

RA-016-D5RA07674A-s001

## Data Availability

The data that support this work are available on request. Supplementary information is available. See DOI: https://doi.org/10.1039/d5ra07674a.
