# Supplementary material for: Synergistic energy harvesting and catalysis in B2SSe–MSSe (M = Mo, W) van der Waals heterostructures
Source: RSC Adv. 2026 Jan 2;16(1):836–48. doi: 10.1039/d5ra07674a (PMC12757770; doi:10.1039/d5ra07674a)
Supplement: RA-016-D5RA07674A-s001 [file RA-016-D5RA07674A-s001.pdf]

Supplementary Information (SI) for RSC Advances.
This journal is © The Royal Society of Chemistry 2025

Supplementary-Information

Synergistic Energy Harvesting and Catalysis in B_2SSe-MSSe (M = Mo, W) van der Waals Heterostructure

Basit Ali[1], Khadija Bashir[2], Yuanping Chen[2], Sailing He[3], M. Idrees[4], B Amin[1*]

[1]*Department of Physics, Abbottabad University of Science & Technology, Abbottabad 22010, Pakistan,* [2]*School of Physics and Electronic Engineering, Jiangsu University, Zhenjiang, 212013, P. R. China,*

[3]*National Engineering Research Center for Optical Instruments, Zhejiang University, Hangzhou 310058, China,*

[4]*School of Chemistry and Chemical Engineering, Shandong University, Jinan, 250100, China*

[*] binukhn@gmail.com,+92-333-943-665

I. COMPUTATIONAL DETAILS

The calculations are performed via Density Functional Theory (DFT) within the framework of the Projector-Augmented-Wave (PAW) potential [1]. The exchange-correlation interactions were treated using the Generalized Gradient Approximation (GGA) in the style of the Perdew-Burke-Ernzerhof (PBE) functional [2], as implemented in the Vienna Ab initio Simulation Package (VASP) [3, 4]. To ensure accurate results, a plane-wave cutoff energy of 550 eV was adopted for all calculations. The convergence threshold for the ionic force was set to 10^{-3} eV/Å , and the self-consistent field (SCF) calculations were converged to an accuracy of 10^{-6} eV per atom. The k-points in the first Brillouin zone were sampled using a $6 \times 6 \times 1$ Monkhorst-Pack grid for geometry optimization and a denser grid of $8 \times 8 \times 1$ for SCF calculations. A vacuum region of 25Å thickness was introduced to eliminate spurious interactions between periodic images along the z-direction. Additionally, the DFT-D3 method was used to accurately capture long-range van der Waals interactions [5]. All atomic positions were fully relaxed until the residual force on each atom was less than 0.01 eV/Å.

The PBE functional within the GGA framework typically underestimates the band gaps of semiconductors [6], therefore the screened hybrid Heyd-Scuseria-Ernzerhof (HSE06) functional was employed to obtain more accurate electronic structures [7]. The effect of spin-orbit coupling (SOC) is substantial in Janus materials; therefore, it is incorporated in all calculations using the second variational method [8]. In this approach, a scalar-relativistic basis set is used to diagonalize the scalar-relativistic part of the Hamiltonian, and then the resulting eigenfunctions are used to construct the full Hamiltonian, including the SOC term. The complete form of the Hamiltonian is:$\hat{H}\psi = \varepsilon\psi + \hat{H}_{\mathrm{SOC}}\psi$, where $\hat{H}_{\mathrm{SOC}}\psi$ denotes the spin-orbit Hamiltonian [9]. Furthermore, the mechanical stability of these systems was verified through calculating the elastic constants using the energy-strain approach [10].

The thermal stability of B_2SSe-MSSe (M = Mo, W) vdWHs are confirmed via ab initio molecular dynamics (AIMD) simulations at 500K using the NVT ensemble (constant number of particles, volume, and temperature) approach, along with the Nose Hoover thermostat to maintain a constant temperature throughout the simulation. The equations of motion were integrated using the velocity Verlet algorithm over a simulation period of 6ps with a time step of 2fs [11, 12]. Meanwhile, the dynamical stability was evaluated via the phonon

spectra using the frozen phonon approach in PHONOPY code based on harmonic interatomic force constants obtained from Density Functional Perturbation Theory (DFPT). To ensure convergence, a $2 \times 2 \times 1$ supercell and a 500 eV cutoff were employed [13, 14].

Using the HSE06 single particle energies and wave functions, the GW_0 approach is employed to calculate the quasiparticle energies. To study the optical spectrum, the HSE06 wave functions are also used to solve the Bethe-Salpeter equation (Tamm-Dancoff approximation). The 10 highest valence and 10 lowest conduction bands are taken into account for calculating the excitonic eigenstates [15].

The photocatalytic response of these materials are investigated by using Mulliken electronegativity in $E_{VBM} = \chi - E_{elec} + 0.5E_g$ and $E_{CBM} = E_{VBM} - E_g$. $E_{CBM}(E_{VBM})$ is conduction(valence) band edge potential, χ is the geometric mean of Mulliken electronegativities of the constituent atoms, E_{elec} = -4.5 eV is the standard electrode potential on the hydrogen scale, and E_g is the bandgap values [16, 17].

II. ACKNOWLEDGMENTS

The authors are grateful to Dr. Julian Evans of Zhejiang University for valuable discussions. The authors would also like to thank Jiangsu University for providing computational support.

[1] W. Kohn and L. J. Sham, Phys. Rev. 140, A1133 (1965)

[2] J. P. Perdew, K. Burke and M. Ernzerhof, Phys. Rev. Lett. 77, 3865 (1996)

[3] G. Kresse and J. Hafner, Phys. Rev. B 47, 558 (1993)

[4] G. Kresse and J. Furthmller, Phys. Rev. B 54, 11169 (1996)

[5] N. Marom, A. Tkatchenko, M. Rossi, V. V. Gobre, O. Hod, M. Scheffler and L. Kronik, J. Chem. Theory Comput. 7, 3944 (2011)

[6] L. S. Cavalcante, M. N. Gjerding, A. Chaves and K. S. Thygesen, J. Phys. Chem. C 123, 16373 (2019)

[7] J. Heyd, G. E. Scuseria and M. Ernzerhof, J. Chem. Phys. 118, 8207 (2003)

[8] X. Ma, X. Wu, H. Wang and Y. Wang, J. Mater. Chem. A 6, 2295 (2018)

[9] M. Idrees, H. U. Din, S. U. Rehman, M. Shafiq, Y. Saeed, H. D. Bui, and B. Amin, Phys. Chem. Chem. Phys. 22(18), 10351 (2020)

[10] R. Golesorkhtabar, P. Pavone, J. Spitaler, P. Puschnig and C. Draxl, Comp. Phys. Commun. 184, 1861 (2013)

[11] J. Zhao, H. Zeng, and G. Yao, Phys. Chem. Chem. Phys. 23(6), 3771 (2021)

[12] Saira, U. Mumtaz, I. Aslam, and M. Sajjad, ACS Appl. Energy Mater. 8, 7, 4404 (2025)

[13] S. Ahmad, K. Sohail, L. Chen, H. Xu, U. H. Din, and Z. Zhou, Int. J. Hyd. Ene. 48(65), 25354 (2023)

[14] S. H. Gul, T. A. Alrebdi, M. Idrees, and B. Amin, Nanoscale Adv. 5(21), 5829 (2023)

[15] B. Amin, N. Singh, U, Schwingenschlogl, Phys. Rev. B 92, 075439 (2015)

[16] J. Liu, X. L. Fu, S. F. Chen, and Y. F. Zhu, Appl. Phys. Lett. 99 191903 (2011)

[17] H. L. Zhuang and R. G. Hennig, Phys. Rev. B 88 115314 (2013)
